# Supplementary material for: Towards a transcriptomic biomarker for the classification of melanocytic neoplasms
Source: PLoS Genet. 2025 Oct 3;21(10):e1011869. doi: 10.1371/journal.pgen.1011869 (PMC12507199; doi:10.1371/journal.pgen.1011869)
Supplement: Attachment — Submitted filename: 2025_7_23_25_KH_Response to reviewers.docx [file pgen.1011869.s003.docx]

**PGENETICS-D-25-00149**

**Towards a transcriptomic biomarker for the classification of melanocytic neoplasms**

**PLOS Genetics**

**Response to reviewers:**

Reviewer #1: This study by Borden and colleagues aims to create a diagnostic tool to better differentiate benign nevi (BN) from malignant cutaneous melanoma (CM). They created transcriptomic datasets from BN and CM and also included publicly available data in their analysis. The authors find that several genes can be used as markers to distinguish BN from CM in their TempO-Seq dataset that are involved in “immune inflammation” pathways. However, they observe batch effects due to duration of sample storage and see differences in gene expression due to gender. Lastly, using publicly available data, they identify a set of genes that may differentiate between CM and BN.

The manuscript is interesting because it investigates transcriptomics as a new diagnostic tool for CM, a malignant condition whose early diagnosis is essential for best outcomes. However, they authors do not perform further studies to confirm their findings (e.g. qPCR, immunohistochemistry, in vitro functional studies), which is a disadvantage of the manuscript. In addition, the differences in gene expression in females and males remain unclear.

Minor points:

•Line 89: You are referring to “from the prior trial”. Can you list, which specific trial you are referring to?

*We have added the specific clinical trial information to the Materials and Methods. NCT00841204, “Sulindac in Preventing Melanoma in healthy Participants Who Are at Increased Risk of Melanoma”*

•Table 2 (*now Table 1*): Can you please add the p-values mentioned from the text (line 219-226) to the table?

*Thank you for the suggestion. We have added these p-values to the table.*

•Line 256: Are any of the differentially expressed genes in females located on chromosome X and if so, which are they?

*A few genes from the X chromosome were identified as differentially expressed in our analysis and are summarized in the table below. RPS4X was significant in males and females, FLNA was significant in males only, and SASH3, GPR143, CYBB, and TCEAL4 were significant in females only. However, consistent with our other analyses, the direction of change between BN and CM was consistent between males and females with a similar magnitude. We have added the following sentence to our results section: “While six X-linked genes were identified as differentially expressed in BN vs. CM (RPS4X in males and females, FLNA in males only, and SASH3, GPR143, CYBB, and TCEAL4 in females only) all of these genes had a consistent direction of change between males and females with a log fold change difference of less than one.”*

| ***Gene*** | ***Direction*** | ***Male log fold change (p-value)*** | ***Females log fold change (p-value)*** |
| --- | --- | --- | --- |
| *RPS4X* | *Down in CM* | *-0.495 (p = 0.0072)* | *-0.665 (p = 0.0021)* |
| *FLNA* | *Up in CM* | *0.699 (p = 0.0496)* | *0.336 (p = 0.34)* |
| *SASH3* | *Up in CM* | *1.701 (p = 0.0689)* | *1.505 (p = 0.00578)* |
| *GPR143* | *Up in CM* | *0.586 (p = 0.394)* | *0.828 (p = 0.0118)* |
| *CYBB* | *Up in CM* | *1.526 (p = 0.140)* | *1.269 (p = 0.025)* |
| *TCEAL4* | *Down in CM* | *-0.201 (p = 0.596)* | *-0.510 (p = 0.038)* |

•Line 279: Did you investigate other reasons for such significant differences in gene expression between the samples? E.g. the thickness of skin sample? Pre-existing conditions? Age of probands?

*Thank you for the question. We revised the manuscript to include other differences between the datasets which may contribute to the observed sex differences in differential expression between BN and MM. All expression data from both the Schartl and TempO-Seq datasets were age-adjusted before differential expression analysis. Schartl did include melanoma samples with a greater depth (up to 10.5 mm). Additionally, the data came from different sequencing technologies (RNA-Seq vs. TempO-Seq). Data for pre-existing conditions was not available for either dataset. Broadly, we observe that the majority of genes show consistency in their differential expression in the analyses segregated by sex, and only a minority have differences between males and females. Given these findings, we do not see convincing evidence for significant sex effects on gene changes during melanoma progression. Further studies with larger datasets may be warranted. We have added a comment on this issue to the text.*

•Line 301-304: Please add the rate-adjusted p-value for all listed pathways.

*Thank you for this suggestion, we have added the false discovery rate-adjusted p-values to Figure 3 and in the figure legend for pathways not included in the figure.*

•Line 323: Please explicitly state why you could not use the TempO-Seq data to build a model.

*While we could have used the TempO-Seq data to create a model that distinguished BN from CM, we were more interested in intermediate lesions that are more difficult to distinguish with current approaches. We clarified this rationale in the text by changing our opening sentence in that paragraph to: “Since the batch effect identified between BN/CM and DN was completely confounded with sample type, we could not move forward with the creation of a model that distinguished intermediate lesions with the TempO-Seq data. Therefore, three available RNA-Seq datasets were obtained including data from BN, PN and CM.”*

•Did you also test the genes and pathways identified from the TempO-Seq data in the publicly available datasets?

*Thank you for your question. We did not redo the differential expression analysis in the publicly available datasets, as this has been done both in the original manuscripts and in our previous manuscript, Borden et al. 2021 (*[*https://pmc.ncbi.nlm.nih.gov/articles/PMC8749980/#app1-cancers-14-00003*](https://pmc.ncbi.nlm.nih.gov/articles/PMC8749980/#app1-cancers-14-00003)*). Notably, our previous study also identified significant enrichment in immune pathways, including cytokine signaling, metal sequestration by antimicrobial peptides, and interleukin-10 signaling. Of the genes in the pathway analysis (Figure 3), SELL, CXCL1, LCP2, and S100A7 showed overlap with the results of the differential expression analyses performed in our previous manuscript. More notably, in the published analysis of differentially expressed genes in BN vs. CM from the Badal dataset, 15 of the 19 genes overlapped with consistent directionality (IFI6, USP18, DLC1, HLA-DRB1, HLA-DRB5, HLA-A, INPP5D, CXCL1, LCP2, CD3E, CD3G, SELL, ITGAL, SERPINA1, and S100A7). We have added a comment on this to the results section following the pathway analysis.*

•I would appreciate confirmatory studies (qPCR, immunohistochemistry, in vitro functional studies) to confirm that these values have an impact in cutaneous melanoma.

*Thank you for the suggestion. We have performed immunohistochemistry (IHC) for proteins encoded by two genes in our transcriptomic biomarker: MMP11 and PYGL. We selected these genes for three reasons: 1) they represented a gene upregulated and a gene downregulated in melanoma with high coefficients and lower variation, 2) they showed consistency in the TempO-Seq data, which contained the samples that we could select for IHC staining, and 3) they had validated antibodies through the Human Protein Atlas.*

*We found that MMP11 exhibited a change in the pattern for expression for CM compared to BN. While BN showed a gradient of staining with greater intensity towards the top and lower intensity towards the bottom of the dermis, the CM displayed uniform staining. This gradient pattern of expression may explain the higher gene expression in CM compared to BN. For PYGL, there was a significant decrease in both the percent and intensity of protein staining in CM compared to BN. These results are summarized in the text and presented in the new Figure 7.*

•Line 396: Are the differences in gene expression cause be storage length or patient gender or both? Please discuss.

*Thank you for the question. The differences in gene expression are much more likely to be caused by storage length than by gender. This is clear from two features of the data. First, as demonstrated in Figure 1A, the major split in the data is caused by the origin of the samples. If sex were the cause of the differences, we would expect to see samples from different origins clustering together based on sex, with a skew in the group distributions. However, what we see is that along Dimension 2 (the y-axis), there is complete separation of the groups based on the origin of the samples alone. Furthermore, the sex distributions were also significantly different between BN and CM, but these clustered together on PCA analysis. We added the following comment to the discussion to clarify the point: “While other demographic characteristics differed between the three groups, only the storage length of the samples is consistent with the complete separation of the BN/CM samples from the DN samples on principal components analysis.”*

•Discussion: What specific confirmational test would you suggest? Do you think that TempO-Seq is a good diagnostic tool for CM?

*Thank you for this question. While we think that TempO-Seq is valuable for model creation, we would suggest that targeted sequencing, quantitative PCR or IHC may be more cost-effective and translatable to the clinic. We have added a sentence to this effect in the discussion.*

Reviewer #2: The authors point out the importance of establishing a transcriptomic biomarker to distinguish between the different melanomas, while also identifying the genetic changes occuring during meningiomagenesis. The authors adjust their analysis based on patient sex and age for each type of nevus, followed by comparing their data to the publically available datasets. Albeit some discrepancies, the study holds novelty and should be considered for further evaluation. This reviewer has some minor comments which should be considered by the authors.

1. On line 216, the authors mention "AN". Please expand on the abbreviation as it is not mentioned prior in the text of the manuscript, only in the legend of table 2 (*now table 1*) as atypical nevi.

*We have ensured that “AN” is defined at its first use in the results section.*

2. The authors mention that the CM samples obtained were from significantly older patients relative to samples obtained from patients with BN and AN. Could the authors comment on this before begining the analysis. Is it known that samples from older patients have different transcription profiles relative to transcriptiomic profiles of CM obtained from younger patients? Please cite relavant articles to this note.

*Thank you, it is known that both sex and age can influence the gene expression profiles in melanoma. We have added a sentence to the results section before the start of the differential expression analysis saying: “Previous work has demonstrated that both age and sex can impact the gene expression profiles of melanoma samples [40]. Therefore, to isolate the effects of the lesion type from the differences in the age and sex distributions between BN and CM, we performed the differential expression analysis separately for males and females and used age-adjusted expression values as described in the methods.”*

3. The authors indicate that there are significant site differences between the different nevi. They should explain if sites need to be comparable for accurate analysis. If the site is indeed of consequence in melanoma, the authors should perform analysis to compare specific sites among the different nevi.

*Thank you, the site has been shown to influence the gene expression profiles in normal skin, and there is some early evidence of its effects in melanoma as well. However, there were no significant differences in the site between BN and CM specimens. We have added an explanation to the results section saying: “While sun exposure has also been identified as influential in gene expression in normal skin [41, 42] and has preliminary evidence of influencing gene expression in melanoma [43], no significant differences in the site were identified between BN and CM specimens. Thus, site was not considered as a covariate in the differential expression analysis.”*

4. The tables should be placed in a chronological order as Table2 is mentioned before Table1.

*Thank you, we have switched the methods to the end in compliance with PLOS Genetics formatting and have placed the tables in chronological order.*

5. In line 278, where the authors mention "since the gene expression differences between the sexes were not......consistent in males and females.". This sentence is extremely confusing and the authors should consider simplifying it for better understanding.

*Thank you for pointing this out. We agree in hindsight that this sentence was not clear. We have revised the last sentence in the section entitled “Differentially expressed genes between BN and CM are consistent in males and females” to: “Since the few gene expression differences observed between sexes were not consistent across datasets, there is limited evidence to suggest a pervasive impact of sex on gene changes through melanoma progression.”*

6. The Log fold changes for the data in figures 2 and 3 is not consistent. The authors look at the differentially expressed genes in CM and BN in the males and females, log change difference cut off is 1, while that for enriched immune pathways is 1.5. Is there a reason for this? To be consistent, the authors might consider including a log change difference cut off of 1.5 or higher for the data in figure2 as well.

*There is no log fold change difference included as a criteria in Figure 2. The “log fold change difference” cut off mentioned here is the difference in the log fold changes between males and females and was included only for the purpose of annotating genes on the plots in Figure 2D-F. We did not include a log fold change cutoff in this figure because we wanted to see the global pattern of consistency (or inconsistency) between the gene expression changes in males and females. However, for the pathway analysis, we focused on the genes most likely to have a substantial biological effect and therefore imposed a log fold change cutoff of 1.5. To clarify what was done, we edited the description in the figure legend of Figure 2 to say: “Genes with a difference in the log fold change (absolute value of the log fold change in males minus the log fold change in females) greater than one between males and females are annotated (range 1.01-3.03).”*

7. The authors point out that there is a "clear separation of the three lession types on line 347 when comparing the genes of PN, CM and BN in a PCA plot. The "clear separation" is that for melanoma and benign, but the PN cluster is more spread in regions with overlap in transcripts from melanoma. Please rephrase this rentence to more accurately represent the data in Figure5C.

-Figure 5C is missing a key.

-Since the number of samples for the PN group is significantly lower relative to malanoma and benign, the "cluster" is not necesasrily defined.

*Thank you for this comment. We rephrased to “Consistent with the original PCA of the data, a PCA restricted to the genes included in the transcriptomic biomarker shows a clear separation of BN and CM with PN occupying an intermediate state (Figure 5C).” We have also added a legend on the figure itself for Figure 5C.*

Reviewer #3: The manuscript by Borden et al. describes the development of a transcriptomic biomarker to distinguish benign nevi (BN), progressing nevi (PN), and cutaneous melanoma (CM). The study addresses an important clinical need and employs multiple datasets and statistical approaches. Overall, the approach is sound, and the results are promising. However, several areas require clarification and expansion to improve readability, rigor, and reproducibility.

Major Points

1. Introduction:

o Line 53-60: The author could add a few sentences describing the key histopathological features that characterize melanocytic lesions ranging from dysplastic nevi to thin (T1a) cutaneous melanoma to make the text more accessible to non-specialists.

*Thank you, we have added the requested information to the introduction.*

o The author could also discuss whether gender influences disease progression.

*Thank you for this suggestion. We have added a comment to the results section, entitled “Differentially expressed genes between BN and CM are consistent in males and females,” discussing the impact of age and sex on expression in melanoma. We selected to add this information to the results section rather than the introduction, because no clear sex differences were identified in our manuscript.*

2. Clarify Batch‐Effect Removal

o Lines 328–329: The text refers to “standard statistical approaches” for batch‐effect removal but does not specify the method. Please provide a brief description of the statistical approach used for batch-effect removal, as details were not provided in the Materials and Methods section (lines 184–185).”

*Thank you for pointing this out. We have added a brief description to the methods section entitled “Creation of the transcriptomic biomarker,” pasted here:*

*“Therefore, we applied an empirical Bayes framework to adjust for batch effects, implemented with the ComBat function from the sva v.3.50.0 package in R [36]. Briefly, expression was standardized across the three datasets, and the mean expression was pooled by the application of a shrinkage estimator using empirical Bayes with parametric priors.”*

3. Validation using multiple datasets

o Lines 273–274: The author should use more than one dataset to investigate whether sex differences exist in these 18 genes between BN and CM.

*Thank you for this comment. While we agree in principle, of the three RNA-Seq datasets that we had available, only the Schartl data was able to be used for this purpose. The Bastian dataset had samples from PN and CM but not BN, preventing an equivalent comparison. The Badal dataset had no BN samples from male patients. Therefore, we were only able to perform this comparison in the Schartl dataset. However, the overwhelming consistency of gene expression changes between males and females and the lack of consistency of the observed differences across datasets suggest that the impact of sex on the BN:MM gene differences is minimal. We have added a comment in the text clarifying this rationale.*

4. Protein‐Level Validation

o Although the transcriptomic biomarker shows consistency across platforms, the functional relevance of these genes would be strengthened by orthogonal (e.g., IHC) validation of at least a subset of these genes at the protein level.

*Thank you for the suggestion. We have performed immunohistochemistry (IHC) for proteins encoded by two genes in our transcriptomic biomarker: MMP11 and PYGL. We selected these genes for three reasons: 1) they represented a gene upregulated and a gene downregulated in melanoma with high coefficients and lower variation, 2) they showed consistency in the TempO-Seq data, which contained the samples that we could select for IHC staining, and 3) they had validated antibodies through the Human Protein Atlas.*

*We found that MMP11 exhibited a change in the pattern for expression for CM compared to BN. While BN showed a gradient of staining with greater intensity towards the top and lower intensity towards the bottom of the dermis, the CM displayed uniform staining. This gradient pattern of expression may explain the higher gene expression in CM compared to BN. For PYGL, there was a significant decrease in both the percent and intensity of protein staining in CM compared to BN. These results are summarized in the text and presented in the new Figure 7.*

5. Inclusion of Dysplastic Nevi (DN)

o Line 395-410: Dysplastic nevi present the greatest diagnostic challenge, yet DN samples were excluded from the TempO-Seq analyses due to batch confounding. Please discuss, if possible, how future studies might prioritize the collection and processing of DN samples to fill this critical gap.

*Thank you, we agree that this is a critical gap. We had a comment on this in the last paragraph of the discussion, but have moved it to the first paragraph of the Discussion, so that it is highlighted as a central consideration in planning future studies aimed at biomarker development for melanoma.*

Figure resubmission:

While revising your submission, please upload your figure files to the Preflight Analysis and Conversion Engine (PACE) digital diagnostic tool, https://pacev2.apexcovantage.com/. PACE helps ensure that figures meet PLOS requirements. To use PACE, you must first register as a user. Registration is free. Then, login and navigate to the UPLOAD tab, where you will find detailed instructions on how to use the tool. If you encounter any issues or have any questions when using PACE, please email PLOS at figures@plos.org. Please note that Supporting Information files do not need this step. If there are other versions of figure files still present in your submission file inventory at resubmission, please replace them with the PACE-processed versions.
